# Burden of Self-Reported Noncommunicable Diseases in 26 Villages of Anand District of Gujarat, India

**DOI:** 10.1155/2015/260143

**Published:** 2015-11-30

**Authors:** Dinesh Kumar, Shyamsundar J. Raithatha, Shanti Gupta, Ravi Raj, Nikhil Kharod

**Affiliations:** ^1^Pramukhswami Medical College, Karamsad, Gujarat 388325, India; ^2^Extension Programmes Department, HM Patel Center for Medical Care and Education, Karamsad, Gujarat 388325, India

## Abstract

*Introduction*. Noncommunicable diseases (NCDs) account for 53% of deaths and 44% of disability adjusted life years lost in India. A survey was undertaken to measure the prevalence of tobacco and alcohol use and self-reported NCDs in a rural community in western part of India.* Methodology*. Trained Village Health Workers did the survey in the years 2012-13 under supervision. The data was collected for five NCDs, namely, hypertension, diabetes mellitus, cancer, heart disease, and mental illnesses.* Results*. 18,269 households with a population of 89755 were covered. Prevalence of any form of tobacco use in the age group of >20 years was 34.5 and 52.7% and 15.2% in males and females, respectively. Prevalence of any NCD was 5.3% with a slightly higher prevalence in females (5.4%) than males (5.2%) in the age group of 20–69 years. Prevalence of NCD multimorbidity (≥2 NCDs) was 0.7% in the age group of 20–69 years. 80.7% of hypertensives and 94.9% of diabetics were taking treatment. More females than males were taking antihypertensive treatment.* Conclusion*. Tobacco use was high. Prevalence of NCDs was less than that reported in other studies. Data generated from this study can be useful in planning a community based NCD programme.

## 1. Introduction

Noncommunicable diseases (NCDs) comprising heart diseases, diabetes, cancer, respiratory diseases, and others contributed to 63% of the 57 million global deaths in 2008 [1]. Eighty percent (80%) of all of these deaths occurred in low- and middle-income countries [1, 2]. These deaths are projected to rise by 15% globally between 2010 and 2020 and with the greatest increases expected to be in low- and middle-income regions [1]. India is undergoing an epidemiological transition with changing patterns of diseases. According to a report, NCDs accounted for a 53% of all deaths and 44% of the disability adjusted life years (DALYs) lost in the year 2005 [3]. The age standardized mortality rate for the NCDs for males and females has been reported to be 757.7 and 537.9 deaths per 100000, respectively [4]. Cardiovascular diseases are the most common diseases contributing to 29% of all the deaths followed by cancer, chronic respiratory diseases, and diabetes which contribute to 7%, 7%, and 2% of the deaths, respectively. As the population of India ages, total number of deaths will increase and the proportion of the deaths due to the chronic diseases will increase as well. The high NCD burden poses a significant challenge to the health care system by accounting for 35% of all outpatient and 40% of inpatient hospitalization bed-days in 2004 [5]. NCDs have a significant effect on the financial condition of households considering the fact that 86.4% of the total expenditure on health care is borne out of pocket in India [6]. According to a World Bank report, the annual income losses to households associated with NCDs account to roughly INR 280 billion (USD 6222 million). It has also been found that 25% of families with a member with CVD experience catastrophic expenditure (health expenditure that is more than 40% of the total nonfood consumption expenditure) [7]. The situation is much worse with cancer, where almost 50% of households with a member with cancer experience catastrophic spending and 25% are driven to poverty by health care expenses [5]. In such a scenario, significant steps are required to be taken at the community level for prevention and control of these diseases. There are very few community based models available in India to address this problem. In order to develop such a model, one of the prerequisites is availability of baseline data on the burden of diseases. It will help in better planning of the resources required for the project. This study reports the findings of a survey related to the burden of self-reported NCDs and tobacco and alcohol use in the community.

## 2. Materials and Methods

Our institute is involved in testing and developing a three-tier health care delivery model with a major focus on noncommunicable diseases. It works in 90 villages and has a network of 5 extension centers (health centers) in the community. A household survey was undertaken in 26 of these villages in the years 2012-13 to identify the catchment population for the programme, estimate the population size for screening of the three cancers, and estimate the burden of tobacco and alcohol use in the population. Moreover data was also collected on prevalence, duration, and treatment status of five self-reported NCDs (hypertension, diabetes mellitus, cancer, heart disease, and mental illnesses). The operational definition for the presence of an NCD was “Whether the doctor has told you that you are suffering from this disease and/or advised treatment for the same?” The survey is repeated annually for updating of the database. The findings of the first survey are presented in this study. The information about all the members of the household was obtained from one of the adult members of the household. If any chronic disease other than these 5 NCDs was present, it was reported as well. The data was collected by Village Health Workers (VHWs) belonging to each of the villages through a one-page questionnaire in local language (Gujarati). The VHWs were females belonging to the village in which they conducted the survey. Their mean age and mean years of schooling were 44.65 years and 9 years, respectively. A one-day training programme comprising didactic sessions and hands on exercises was organised for the VHWs for the data collection process. The training was extended on field as well where the VHWs did the data collection under supervision in the initial phase. The process of data collection lasted from September 2012 to December 2013. The data collection process was supervised by 3 Field Supervisors (FS) and 3 Programme Executives (PEs). The FS checked all the forms for completeness and 10% for quality of data collection by cross verification through home visits. The PEs verified 5% of the forms for completeness and 5% of the forms for quality of data collection by cross verification through home visits. The data was entered in a data entry software designed specifically for this project, by five data entry operators. The PEs checked 5% of the entered forms for accuracy and 5% for completeness during the data entry process. A daily backup and online storage process through Dropbox was established to prevent loss of data. Moreover postentry data quality checks were run through Statistical Package for Social Science (SPSS) 16 wherein a descriptive analysis was done to identify outliers which were cross-checked with the hard copies of the forms. Ethical clearance was obtained from the institutional Human Research Ethics Committee (HREC) for publishing the findings of the survey.

*Analysis*. Age-sex pyramid was created. Proportions were calculated for self-reported prevalence of five NCDs. Crude as well as age adjusted prevalence was calculated. The prevalence was adjusted by direct standardization method using the standard WHO population [8]. Chi-square test was applied for testing statistical significance of difference in the prevalence of NCDs between different groups.

## 3. Results

There were 18,269 households contributing to a population of 89755 with an average family size of 4.91. 70.2% of the population comprised adults >20 years of age (Table 1). The overall gender ratio and the gender ratio in the 0–6 years' age group were 914 and 894 females per 1000 males, respectively. Agriculture (46.8%) was the major occupation for heads of the households followed by labour work in nonagricultural jobs (22%). 34.7% of the population (age group 20–69 years) reported at least some form of tobacco (oral tobacco, beedi, and cigarettes) or alcohol use. Similarly 2.5% of the population in the age group of 5–19 years (children) reported at least some form of tobacco or alcohol use. The prevalence of tobacco and alcohol use among males was significantly higher than that among females (Table 2). The prevalence of any NCD in the population was 5.3% and it was slightly higher in females 5.4% as compared to males 5.2% (Table 3). 80.7% of hypertensives and 94.9% of the diabetics were taking treatment. The proportion of hypertensive females (82.5%) taking treatment was significantly higher than that for males (78.3%) with a *p* value of 0.003. In case of diabetes, this difference was not significant. The prevalence of NCDs increased with the age (*p* < 0.001, chi-square for trend) except for mental illnesses. Increasing educational status also showed association with prevalence of hypertension and diabetes mellitus (*p* < 0.001, chi-square for trend). Substance use did not show any statistical association with self-reported NCD prevalence. The details for association of age, education, and substance use categories are provided in Table 4. The prevalence of any NCD and NCD multimorbidity (≥2 NCDs [9]) in the population of >20 years of age was 4.5% and 0.7%, respectively (Table 5). The mean age at diagnosis of hypertension and diabetes mellitus in our study was 49.9 (SD-9.0) and 49.2 (SD-9.6) years, respectively (Table 6). It was significantly higher in those who were illiterate as compared to those who had more than 8 years of schooling (primary education).

## 4. Discussion

**Figure 1:**
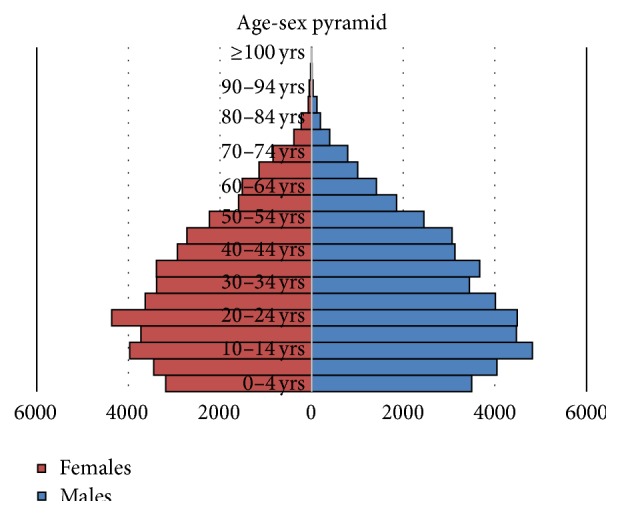
Population pyramid for the study population.

This survey conducted by trained Village Health Workers reports prevalence of self-reported NCDs and substance use patterns (tobacco and alcohol) in a rural population of the Gujarat state located in the western part of India. The adult gender ratio and the child gender ratio (0–6 years) reported in the study were 914 and 894 females per 1000 males, respectively. Similar figures for Anand District, Gujarat, and India were 925 and 884, 919 and 890, and 943 and 919, respectively [10]. The age-gender pyramid (Figure 1) shows bulge in the 10–25 years' group compared to the wide base in under 5 years' age group seen in the developing countries. This probably indicates the demographic transition through which the community is passing.

The prevalence of tobacco use in the age group of 20–69 years was found to be 34.5% (52.7% and 15.2% in males and females, resp.). The prevalence of oral tobacco use was 27.7% (39.8% and 14.8% in males and females, resp.). As per the National Family Health Survey 3 (NFHS 3), the prevalence of any form of tobacco use in the age group of 15–49 years was found to be 60.2% in rural Gujarat with prevalence of 63.4% and 11.3% for males and females, respectively. The prevalence of oral tobacco consumption was found to be 45.2% and 6% among males and females, respectively. However, the data was based on the responses of 737 male participants and 2114 female participants only. Also NFHS 3 had preceded our survey by about 6 years during which the prevalence might have changed [9].

The prevalence of any NCD was 5.3% in the age group of 20–69 years (5.4% in males and 5.2% in females). In a multisite study conducted in 5 Asian countries in 2005, 22.7% of men and 31.6% of women in the age group of 25–64 years reported having at least 1 of the chronic health conditions of interest. 5.1% of men and 9.2% of women reported having 2 or more chronic conditions. Women had more NCDs than men, the prevalence of NCDs increased with age, and people with the least education were more likely to have chronic conditions [11]. In the WHO-SAGE study conducted on nationally representative data in India, 28.5% of the sample population had at least one NCD and 8.9% had NCD multimorbidity. The prevalence of multimorbidity increased from 1.3% in 18–29-year-olds to 30.6% in those aged 70 years and above [12]. The reasons for low prevalence reported in our study could be multiple such as information collected from a representative of the household, rural population having lower burden of the disease, or greater portion of the disease being undiagnosed. The prevalence of any NCDs and that of hypertension, diabetes mellitus, and heart disease increased with the increasing age as observed in other studies in India and outside as well [11–13]. The difference in the prevalence of any NCDs in males and females was not significant in our study. The prevalence for any NCD and hypertension was higher in the illiterate groups than that in the group having education of ≤8 years of schooling. Thereafter it increased progressively with increasing education. Such a phenomenon was not observed for other NCDs.

The prevalence of hypertension reported in our study was 4.2% with 3.7% and 4.4% prevalence reported in males and females, respectively (Table 3). It has been reported to be in the range between 20–40% in urban adults and 12–17% among rural adults [14]. In the multicountry study conducted in the year 2005, it was reported to be 7.4% across all the sites and 3.2% in the Indian site which is comparable with our finding [12]. 80.7% of all the hypertensive patients in our study were receiving treatment for their disease status. In a systematic review, the pooled estimate for the percentage of hypertensive patients being treated was found to be 24.9% (16.7–33.0) [15] which was quite low as compared to that reported in our study. This could be due to the fact that majority of those who reported to be suffering from hypertension in our study reported so because they were taking treatment while there might be a significant portion who might be suffering from hypertension but not taking regular treatment or taking irregular treatment and might not have reported the same.

The prevalence of diabetes in the adult population (>20 years) in our study was 1.5%. As per the 6th Edition (2013) of the International Diabetes Federation Atlas, it is estimated that around 50% of the diabetics are not aware of their condition; that is, they are not diagnosed [16]. Considering this fact, the actual prevalence of diabetes in our population can be estimated to be around 3.5%. The multicountry study reported prevalence of 2.2% across all the sites and 1.7% in India [11]. The ICMR India Diabetes (ICMR-INDIAB) study reported prevalence of 2.3–5.2% for diabetes in rural parts of 4 states of India by conducting diagnostic tests on the population, which is comparable with our estimates [17]. However, in the WHO-ICMR Indian NCD risk factor surveillance study, the prevalence of self-reported diabetes mellitus was found to be 3.1% in a sample of 13,524 individuals (age group: 15–64 years) from different geographical locations in the country [18]. The mean age at diagnosis for diabetes mellitus in our study was 49.2 (SD-9.6) years. In a nationwide survey conducted in 2011 for 6168 patients taking treatment at 330 centers comprising general hospitals, diabetes clinics, and referral clinics in India, the mean age at diagnosis was reported to be 45.4 (±10.9) [19]. However it was a predominantly urban population, reflecting a greater awareness and a greater access to health care services as compared to our study population which was in a rural setting. The higher mean age at diagnosis for hypertension and diabetes mellitus in our study for those who were illiterate as compared to those with more than 8 years of schooling reflects the effect of education on awareness about the need for screening and early diagnosis and access to health care services.

The prevalence of heart disease in the study population was found to be 0.6%. The estimated prevalence of coronary heart disease is around 3-4% in rural areas and 8–10% in urban areas among adults older than 20 years [20].

The self-reported prevalence of chronic conditions is subject to recall bias and may not reflect the true prevalence. Reporting also can be affected by respondents' knowledge, manifestations of the illness in everyday life, their willingness to report the condition, and frequency of contact with a physician [21], although it has been found in studies conducted in Western countries that self-reports of chronic conditions are quite accurate when compared with physician diagnoses [22–24]. Due to different sociodemographic characteristics of our population and the study design followed in our population, the self-reported prevalence might be reported to be lower as compared to other studies [11, 15, 17].

## 5. Conclusions

Prevalence of NCDs in our study was found to be less than that reported in other studies. The prevalence increased with age and there was no significant difference in the prevalence in males and females. Effect of education on the age of diagnosis of hypertension and diabetes mellitus could be documented in the study. The percentage of hypertensives and diabetics taking treatment was significantly high with more females taking treatment for hypertension as compared to males. Data generated from this study can be useful in planning a community based NCD programme. It provides the much needed population denominator which can be utilised to calculate various incidence, prevalence, and mortality rates which can serve as impact indicators for various community based programmes. Moreover it provides information on the number of patients of NCDs which are in need of treatment. It is useful information for calculating the need of resources and better planning of the programme related activities. Further studies may be undertaken to identify the actual burden of these diseases through more objective assessments and investigation by trained paramedical personnel. High prevalence of tobacco use calls for developing community based tobacco control and cessation activities.

## Figures and Tables

**Table 1:** Age and sex distribution and education status of the study population.

Variable	Frequency	Percent
Total population	89755	100%
Age groups		
Children (0–9 years)	16067	17.9%
Adolescents (10–19 years)	15098	16.8%
Adults (≥20 years)	58590	65.2%
Elderly (>60 years)	8288	9.2%
Sex		
Male	46903	52.3%
Female	42852	47.7%
Education status in adults (aged 20–69 years)		
Illiterate	10372	18.7%
≤8 years of schooling	21547	38.9%
8–12 years of schooling	29852	35.8%
>12 years of schooling	3680	6.6%

**Table 2:** Prevalence of substance abuse in the total population and genderwise (in adults aged 20–69 years).

Substance abuse	Total prevalence (%)	Prevalence in females (%)	Prevalence in males (%)	*p* value^*∗*^ (chi-square test)
Any	34.7	15.2	53.1	<0.001
Any tobacco	34.5	15.2	52.7	<0.001
Oral tobacco	27.7	14.8	39.8	<0.001
Beedi	9.3	0.5	17.6	<0.001
Cigarette	0.4	0	0.7	<0.001
Alcohol	2.1	0.2	3.9	<0.001
Tobacco + alcohol	0.6	0.000	1.1	<0.001

^*∗*^ *p* value is for difference between genders and is based on chi-square test.

**Table 3:** Prevalence of self-reported noncommunicable diseases (NCDs) in the adult population (aged 20–69 years).

NCD	Age adjusted prevalence^*∗*^	Crude prevalence	Prevalence in males	Prevalence in females	*p* value^#^ (Chi-square test)
Hypertension	4.2%	3.7%	3.7%	4.4%	<0.001
Diabetes mellitus	1.5%	1.3%	1.5%	1.2%	0.003
Cancer	0.1%	0.1%	0.1%	0.1%	0.090
Heart disease	0.44	0.39%	0.5%	0.3%	<0.001
Mental illnesses	0.3%	0.3%	0.3%	0.3%	0.264
Any disease	5.9%	5.3%	5.2%	5.4%	0.690

^*∗*^Adjusted with WHO standard population [8].

^#^For crude prevalence between males and females.

**Table 4:** Prevalence of self-reported noncommunicable diseases in adults (20–69 years) compared with respect to various sociodemographic variables.

Variable	Any NCD	Hypertension	Diabetes mellitus	Heart disease	Cancer	Mental illness
Age groups						
20–40 years	389 (1.3%)	209 (0.7%)	53 (0.2%)	22 (0.1%)	12 (0.1%)	77 (0.3%)
40–60 years	1571 (7.9%)	1135 (5.7%)	428 (2.1%)	127 (0.6%)	27 (0.1%)	66 (0.3%)
60–69 years	993 (19.5%)	748 (14.7%)	264 (5.2%)	71 (1.4%)	20 (0.4%)	20 (0.4%)
Education						
Illiterate	571 (5.5%)	390 (3.8%)	72 (0.7%)	35 (0.3%)	5 (0.0%)	57 (0.5%)
≤8 years of schooling	1057 (4.9%)	760 (3.5%)	231 (1.1%)	88 (0.4%)	31 (0.1%)	54 (0.3%)
8–12 years of schooling	1083 (5.5%)	778 (3.9%)	364 (1.8%)	81 (0.4%)	19 (0.1%)	45 (0.2%)
>12 years of schooling	242 (6.6%)	164 (4.5%)	78 (2.1%)	16 (0.4%)	4 (0.1%)	7 (0.2%)
Substance use						
Oral tobacco	629 (4.1%)	464 (3%)	144 (0.9%)	56 (0.4%)	16 (0.1%)	41 (0.3%)
Smoking	442 (8.4%)	263 (5%)	84 (1.6%)	48 (0.9%)	12 (0.2%)	17 (0.3%)
Oral tobacco and smoking	982 (5.1%)	670 (3.5%)	213 (1.1%)	94 (0.5%)	24 (0.1%)	51 (0.3%)
Alcohol	56 (4.8%)	33 (2.8%)	9 (0.8%)	9 (0.8%)	1 (0.1%)	4 (0.3%)
Any addiction	987 (5.1%)	672 (3.5%)	214 (1.1%)	96 (0.5%)	24 (0.1%)	53 (0.3%)
All three	12 (3.7%)	8 (2.5%)	1 (0.3%)	2 (0.6%)	0 (0%)	0 (0%)

**Table 5:** Association between number of NCDs with other variables (age group 20–69 years).

Variable	Number of NCDs		
	0	1	≥2
Overall (for adults >20 years)	52576 (94.8%)	2152 (4.5%)	363 (0.7%)
Age groups			
20–39 years	30043 (98.9%)	333 (1.1%)	13 (0.0%)
40–59 years	18388 (92.1%)	1401 (7.0%)	184 (1.0%)
60–69 years	4145 (81.5%)	778 (15.3%)	166 (3.3%)
Gender (for adults >20 years)			
Male	27134 (95.1%)	1214 (4.3%)	189 (0.7%)
Female	25442 (94.5%)	1298 (4.8%)	174 (0.6%)
Education (for adults >20 years)			
Illiterate	9851 (95%)	486 (4.7%)	35 (0.3%)
≤8 years of schooling	20517 (95.2%)	915 (4.2%)	115 (0.5%)
8–12 years of schooling	18760 (94.5%)	914 (4.6%)	178 (0.9%)
>12 years of schooling	3448 (93.7%)	197 (5.4%)	35 (1%)
Substance use			
Oral tobacco	14726 (96%)	538 (3.5%)	83 (0.5%)
Smoking	4894 (93%)	323 (6.1%)	45 (0.9%)
Oral tobacco and smoking	18197 (95.2%)	797 (4.2%)	116 (0.6%)
Alcohol	1111 (95.8%)	42 (3.6%)	7 (0.6%)
Any substance	18337 (95.2%)	804 (4.2%)	116 (0.6%)
All three	315 (96.6%)	11 (3.4%)	0

**Table 6:** Self-reported age at diagnosis of hypertension and diabetes mellitus in adults (20–69 years) with respect to various sociodemographic variables.

Variable	Mean age at diagnosis of hypertension in years (SD)	Mean age at diagnosis of diabetes mellitus in years (SD)
Overall	49.9 (9.0)	49.2 (9.6)
Age groups		
20–39 years	31.0 (5.8)	30.3 (4.7)
40–59 years	46.4 (6.1)	46.2 (6.0)
60–69 years	57.0 (5.5)	58.5 (4.9)
Gender		
Male	50.3 (9.0)	48.8 (9.1)
Female	49.7 (10.0)	49.7 (9.2)
Education		
Illiterate	51.9 (9.5)	52.4 (9.6)
≤8 years of schooling	50.3 (9.8)	49.3 (10.0)
8–12 years of schooling	48.9 (11.0)	48.4 (10.9)
>12 years of schooling	48.7 (9.9)	50.0 (7.4)
Substance abuse		
Oral tobacco	50.3 (10.0)	49.8 (9.0)
Smoking	51.5 (10.0)	49.3 (9.6)
Alcohol	52.9 (6.7)	49.4 (13.4)
Oral tobacco and smoking	50.9 (9.5)	49.7 (9.3)
Any	52.8 (7.8)	49.7 (9.3)
All three	50.8 (9.5)	49.2 (9.3)
Number of NCDs		
1	49.7 (9.8)	47.9 (9.4)
2	51.2 (8.5)	51.5 (7.9)
≥3	50.1 (9.2)	49.4 (10.2)

## References

[1] World Health Organization (2011). *Global Status Report on Noncommunicable Diseases*.

[2] World Health Organization Global burden of disease: 2004 update. http://www.who.int/healthinfo/global_burden_disease/2004_report_update/en/.

[3] Srinath Reddy K., Shah B., Varghese C., Ramadoss A. (2005). Responding to the threat of chronic diseases in India. *The Lancet*.

[4] WHO (2011). *Noncommunicable Diseases Coutnry Profiles*.

[5] Mahal A., Karan A., Engelgau M. (2010). *The Economic Implications of Non-Communicable Diseases for India*.

[6] National Health Accounts Cell National health accounts India 2004-05. http://planningcommission.nic.in/reports/genrep/health/National_Health_Account_04_05.pdf.

[7] World Health Organization (2000). *Health Systems: Improving Performance*.

[8] Ahmad O. R., Boschi-Pinto C., Lopez A. D., Murray C. J., Lozano R., Inoue M. (2001). *Age Standardization of Rates: A New WHO Standard*.

[9] http://www.rchiips.org/nfhs/NFHS-3%20Data/gujarat_state_report_for_website.pdf.

[10] http://www.census2011.co.in/census/district/196-anand.html.

[11] Van Minh H., Ng N., Juvekar S. (2008). Self-reported prevalence of chronic diseases and their relation to selected sociodemographic variables: a study in INDEPTH Asian sites, 2005. *Preventing Chronic Disease*.

[12] Basu S., King A. C. (2013). Disability and chronic disease among older adults in India: detecting vulnerable populations through the WHO SAGE Study. *American Journal of Epidemiology*.

[13] Pati S., Agrawal S., Swain S. (2014). Non communicable disease multimorbidity and associated health care utilization and expenditures in India: cross-sectional study. *BMC Health Services Research*.

[14] Gupta R. (2004). Trends in hypertension epidemiology in India. *Journal of Human Hypertension*.

[15] Anchala R., Kannuri N. K., Pant H. (2014). Hypertension in India: a systematic review and meta-analysis of prevalence, awareness, and control of hypertension. *Journal of Hypertension*.

[16] International Diabetes Federation (2013). *IDF Diabetes Atlas*.

[17] Anjana R. M., Pradeepa R., Deepa M. (2011). Prevalence of diabetes and prediabetes (impaired fasting glucose and/or impaired glucose tolerance) in urban and rural India: phase i results of the Indian Council of Medical Research-INdia DIABetes (ICMR-INDIAB) study. *Diabetologia*.

[18] Mohan V., Mathur P., Deepa R. (2008). Urban rural differences in prevalence of self-reported diabetes in India—the WHO-ICMR Indian NCD risk factor surveillance. *Diabetes Research and Clinical Practice*.

[19] Mohan V., Shah S. N., Joshi S. R. (2014). Current status of management, control, complications and psychosocial aspects of patients with diabetes in India: results from the DiabCare India 2011 Study. *Indian Journal of Endocrinology and Metabolism*.

[20] Ghaffar A., Reddy K. S., Singhi M. (2004). Burden of non-communicable diseases in South Asia. *British Medical Journal*.

[21] Dalstra J. A. A., Kunst A. E., Borell C. (2005). Socioeconomic differences in the prevalence of common chronic diseases: an overview of eight European countries. *International Journal of Epidemiology*.

[22] Kriegsman D. M. W., Penninx B. W. J. H., Van Eijk J. T. M., Boeke A. J. P., Deeg D. J. H. (1996). Self-reports and general practitioner information on the presence of chronic diseases in community dwelling elderly. A study on the accuracy of patients' self-reports and on determinants of inaccuracy. *Journal of Clinical Epidemiology*.

[23] Goldman N., Lin I.-F., Weinstein M., Lin Y.-H. (2003). Evaluating the quality of self-reports of hypertension and diabetes. *Journal of Clinical Epidemiology*.

[24] Kehoe R., Wu S.-Y., Leske M. C., Chylack L. T. (1994). Comparing self-reported and physician-reported medical history. *American Journal of Epidemiology*.

